# Modeling the epigenetic attractors landscape: toward a post-genomic mechanistic understanding of development

**DOI:** 10.3389/fgene.2015.00160

**Published:** 2015-04-23

**Authors:** Jose Davila-Velderrain, Juan C. Martinez-Garcia, Elena R. Alvarez-Buylla

**Affiliations:** ^1^Departamento de Ecología Funcional, Instituto de Ecología, Universidad Nacional Autónoma de MéxicoMexico City, Mexico; ^2^Centro de Ciencias de la Complejidad (C3), Universidad Nacional Autónoma de MéxicoMexico City, Mexico; ^3^Departamento de Control Automático, Cinvestav-Instituto Politécnico NacionalMexico City, Mexico

**Keywords:** GRN, epigenetic landscape, attractors, cell-fate, morphogenesis, stem-cells, cancer

## Abstract

Robust temporal and spatial patterns of cell types emerge in the course of normal development in multicellular organisms. The onset of degenerative diseases may result from altered cell fate decisions that give rise to pathological phenotypes. Complex networks of genetic and non-genetic components underlie such normal and altered morphogenetic patterns. Here we focus on the networks of regulatory interactions involved in cell-fate decisions. Such networks modeled as dynamical non-linear systems attain particular stable configurations on gene activity that have been interpreted as cell-fate states. The network structure also restricts the most probable transition patterns among such states. The so-called Epigenetic Landscape (EL), originally proposed by C. H. Waddington, was an early attempt to conceptually explain the emergence of developmental choices as the result of intrinsic constraints (regulatory interactions) shaped during evolution. Thanks to the wealth of molecular genetic and genomic studies, we are now able to postulate gene regulatory networks (GRN) grounded on experimental data, and to derive EL models for specific cases. This, in turn, has motivated several mathematical and computational modeling approaches inspired by the EL concept, that may be useful tools to understand and predict cell-fate decisions and emerging patterns. In order to distinguish between the classical metaphorical EL proposal of Waddington, we refer to the *Epigenetic Attractors Landscape* (EAL), a proposal that is formally framed in the context of GRNs and dynamical systems theory. In this review we discuss recent EAL modeling strategies, their conceptual basis and their application in studying the emergence of both normal and pathological developmental processes. In addition, we discuss how model predictions can shed light into rational strategies for cell fate regulation, and we point to challenges ahead.

## 1. Introduction

The progressive loss of potency from pluripotent stem cells to mature, differentiated cells, as well as the reproducible emergence of spatiotemporal patterns through the course of development has been always perceived as strong evidence of the robustness and *deterministic* nature of development. The explanation of such a robust process has puzzled researchers for many years. For a long time, although not always stated explicitly, the prevailing paradigm in developmental biology was supported on two fundamental paradigms: (1) a mature cell, once established, displays an essentially irreversible phenotype; and (2) the developmental process is controlled by a “program” as a genomic blueprint following a simplistic linear scheme of causation in an essentially deterministic fashion. Experimental and theoretical studies in the last decade have challenged these assumptions. It has been shown that a differentiated state of a given cell is not irreversible as previously thought, and that in fact, it is possible to reprogram differentiated cells into pluripotent states with a plethora of protocols in plants and animals (Grafi, 2004; Takahashi and Yamanaka, 2006; Takahashi et al., 2007; González et al., 2011).

Overall, a growing body of empirical evidence now supports intrinsic physical processes as a fundamental source of order instead of deterministic pre-programmed rules (Huang, 2009; Mammoto and Ingber, 2010). Although these observations have just recently shift the focus of study in developmental biology and biomedical research, the new evidence is in line with the proposals that early theoretical biologists posited decades ago (see, for example Waddington, 1957; Goodwin, 1963; Kauffman, 1969, 1993; Goodwin, 2001). C. H. Waddington was one of the first to point out that the physical implementation of the information coded in the genes and their interactions imposes developmental constraints while forming an organism. Waddington's heuristic model of the epigenetic landscape (EL) was a visionary attempt to consolidate these ideas in a conceptual framework that enables the discussion of the relationship between genetics, development, and evolution in an intuitive manner. Waddington's proposal was inspired in a formal dynamic systems approach, nonetheless (Waddington, 1957; Gilbert, 1991; Slack, 2002).

Nowadays in the data-rich, post-genomic era the EL has been consolidated as a useful conceptual model for the discussion of the mechanistic basis underlying cellular differentiation—particularly trans-differentiation and reprogramming events (Alvarez-Buylla et al., 2008; Enver et al., 2009; Fagan, 2012; Ladewig et al., 2013). This field has become particularly active due to its potential medical applications using stem cells systems biology as a means for discovering efficient reprogramming or therapeutic strategies by combining mathematical and computational modeling with experimental techniques (MacArthur et al., 2008, 2009; Roeder and Radtke, 2009; Huang, 2011; Zhou and Huang, 2011). Recently, though, numerous critiques to Waddington's original model have been presented in light of the dynamical plasticity of differentiated cells (see, for example Balázsi et al., 2011; Ferrell, 2012; Furusawa and Kaneko, 2012; Garcia-Ojalvo and Arias, 2012; Sieweke, 2015). In this review, we claim that the formalization of the EL in the context of the study of the dynamical properties of GRNs enables a formal framework which provides the necessary flexibility for a model to be both: (1) consistent with the observed inherent plasticity of developing cells and (2) formally derived from the uncovered regulatory underpinnings of cell-fate regulation. It is thus important to note that this GRN associated EL model is not to be confused with the literal, metaphorical model presented by Waddington, which some authors have associated only to the static diagrammatic proposal originally put forward (West-Eberhard, 2003). In order to highlight such distinction, here we will refer to the EL model associated with the dynamics of GRNs as the *epigenetic attractors landscape* (EAL).

The conceptual distinction between the classical EL and the EAL proposed here, as well as its relevance as a consistent model for the prevailing theories of differentiation is going to be exposed by the authors elsewhere. In this contribution we instead focus on the mathematical approaches which have been developed to derive an EAL as an extension of the conventional dynamical analyses of experimentally grounded GRN models. Importantly, we deliberately use the generic term EAL to refer to a group of dynamical models which are quite diverse in mathematical properties and structure, however we do so for phenomenological reasons: all the approaches try to formally tackle the phenomenon of cellular differentiation taking the classical EL model as a conceptual basis. Given the current relevance of such a modeling exercise applied to molecular networks involved on processes such as stem cell differentiation (Li and Wang, 2013), tissue morphogenesis (Alvarez-Buylla et al., 2010), and carcinogenesis (Choi et al., 2012; Wang et al., 2014); and the fact that different approaches have been proposed in order to reach similar goals (Huang, 2009, 2012; Zhou et al., 2012), we hope that the present integrative review may prove useful for a wide range of biological applications. Our main objective is 2-fold: (1) to help different research groups attempting to formalize the EAL to reduce the gap existing between current different approaches and (2) to contribute to shape a common and formal discussion ground on EAL models among experimentalists and theoretical biologists. Accordingly, we have decided to favor conceptual clarity over technicalities through the text, and to point to original references where more detail is available if necessary. We apologize for the theoretically oriented reader for the lack of mathematical formality.

### 1.1. The dynamical-systems view of cell biology

**Figure 1:**
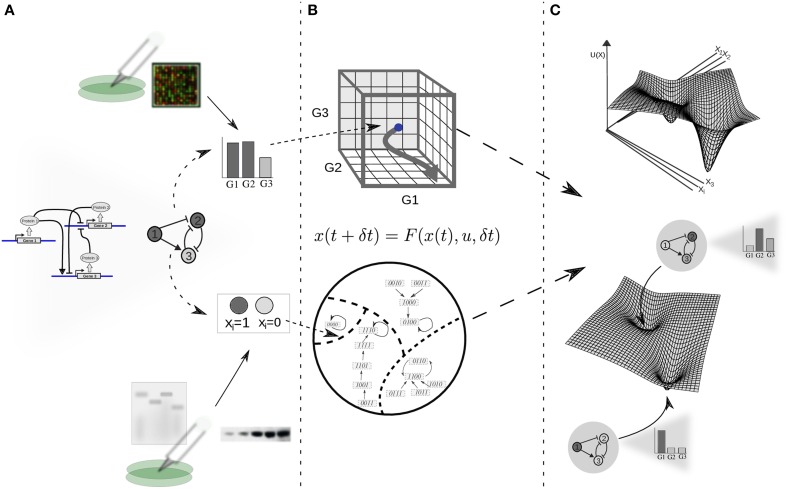
**From experimental data on gene function and interactions to a dynamic gene regulatory network and epigenetic landscape model. (A)** The architecture of a GRN is proposed given available experimental molecular data; the state of the network is specified as a gene expression profile or gene on/off (1/0) configuration for the case of continuous or discrete state models, respectively. Boolean or differential equations are used, respectively. **(B)** The complete set of possible states define a continuous (above) or discrete (below) state space, where each state corresponds to a point; changes in gene expression during developmental dynamics manifest as trajectories in this abstract space (here depicted as arrows). **(C)** In an intuitive characterization of an EL, an “elevation” value *U*(*x*) is associated to each network state *x*. The association of “elevation” values to network states, or more generally, the quantitative characterization of their relative stability is the ultimate goal of EL modeling efforts. For illustrative purposes, the EL is depicted here as a hypothetical low-dimensional projection.

The modern picture of the EL is framed in the context of GRN dynamics (Kauffman, 1969; Mendoza and Alvarez-Buylla, 1998; Huang, 2012), and its theoretical basis is a dynamical-systems perspective. From here on we will refer to this view of the EL model as the EAL. Under dynamical-systems framework a cell is considered a dynamical system, assuming that its state at a certain time can be described by a set of time-dependent variables. As a first approximation, it is commonly assumed that the amount of the different proteins within the cell or, for practical reasons, the levels of gene expression (i.e., expression profiles) are sufficient to describe such state (Huang, 2013). Thus, the expression profile is conventionally taken as the set of variables representing the state of the cell; each gene in the cell's GRN representing one variable (see Figure 1). Mathematically, the set of variables is represented as a state vector given by **x**(*t*) = [*x*_1_(*t*), *x*_2_(*t*) …, *x_n_*(*t*)] for a GRN with *n* genes. Given such specification, it is useful to imagine an abstract space termed the *state space* of the system. In the context of GRNs the state space comprises all the theoretically possible states a cell can exhibit; each point in this abstract space represents one particular expression profile (Figure 1B). Furthermore, it is assumed that the cell state at a certain time and the cell state at a later time are connected by a state trajectory in a causal way.

Mathematically, the current cell state is a function or a more general mapping of the initial state and certain additional parameters. The connection between cell states can be formally expressed by a dynamical equation,

(1) x(t+δt)=F(x(t),u,δt),

where **F** represents the map that connects one state with the immediately previous sate (**F** is also known as the *transition map*), **x**(*t*) denotes the state at a certain time *t*, and **u** stands for the vector of additional parameters. Both the time increments δ*t* and the state variables *x_i_*(*t*) can be either continuous or discrete, depending on the chosen mathematical formalism. Within the cell, the map **F** is implemented by the architecture of the GRN, which specifies both the topology of the network and the nature and form of the corresponding gene regulations (Huang, 2009). Because of globally conditioned gene behavior due to mutual gene regulatory interactions, through the causal connections between cell states, the GRN imposes dynamical constraints and limits the permissible behavior of the cell. Of special interest are the transient and emergent stable configurations that the cell may attain as a result. The existence of the dynamical map **F** expresses the causality of the cellular developmental process and the mechanistic character of GRN dynamical models.

One of the most salient and impressive features of GRNs is the existence of a small number of stationary or quasi-stationary gene configurations within the state space (Kauffman, 1969). Given a specific GRN, a set of cell states satisfy the constraints imposed by the GRN; that is, each of these cell states is connected to itself by the map **F** (i.e., x^*^ = **F**(x^*^, **u**)). When these steady states (x^*^) are also resilient to perturbations, that is, if they return back to the steady state after being kicked away by state variations either of intrinsic or external origin, we refer to them as *attractors*. In the case of quasi-stationary states, if a set comprised of several individual states repeats in a cyclic manner it corresponds to a cyclic attractor. All other states are either unstable or form part of transitory trajectories channeled toward one of these attractor states. The theory posits that attractor states correspond to the observable robust cell phenotypes, cell types, or cellular processes; and that these emerge as a natural consequence of the dynamical constraints imposed by the underlying GRN (Huang and Kauffman, 2009; Huang, 2013). For a more formal definition of attractors in dynamical systems theory see (Fuchs, 2013a).

### 1.2. Extending GRN to EAL models

The postulation of experimentally grounded GRN dynamical models, their qualitative analysis and dynamical characterization in terms of control parameters, and the validation of predicted attractors against experimental observations has become a well-established framework for the study of developmental dynamics in systems biology—see, for example: (Mendoza and Alvarez-Buylla, 1998; Von Dassow et al., 2000; Albert and Othmer, 2003; Espinosa-Soto et al., 2004; Huang et al., 2007; Graham et al., 2010; Sciammas et al., 2011; Hong et al., 2012; Jaeger and Crombach, 2012; Azpeitia et al., 2014). The qualitative analysis of the dynamics of GRN models is well-suited for the study of the specification of cell fates as a result of the constrains imposed by the associated GRN. This conventional analysis includes the identification and local characterization of attractor states, and the comparison of these predicted cell-type configurations with the ones that are actually observed in the corresponding biological system (Figures 1A,B).

If one is interested in studying the potential transition events among the already characterized stable cellular phenotypes, however, several difficulties arise. Standard analysis of dynamical systems, which focuses on the existence and local properties of a given attractor, fail to capture the main problem which is concerned with the relative properties of the different attractors (Zhou et al., 2012). In deterministic GRN models, given certain values for the related control parameters, the system under study always converges to a single attractor if initialized from the same state, and once it attains such steady-state it remains there indefinitely. In contrast, during a developmental process, cells change from one stable cell configuration to another one in specific temporal and spatial or morphogenic patterns. Additional formalisms are needed in order to explore questions regarding how cells in the course of differentiation transit among available given attractors, or the order in which the system converges to the different attractors given an initial condition; as well as to predict how these mechanisms can be altered by rational strategies.

#### 1.2.1. EAL modeling goals

The need for extending GRN dynamical models beyond standard local analysis is related with the interest in addressing the following—and similar—questions. Conceptually, given an experimentally determined GRN, how can we explain and predict both specific “normal” and altered cellular differentiation events or morphogenic patterns? Is it possible to control the fate of differentiation events through well-defined stimuli? Can we deliberately cause altered morphogenic patterns by means of either genetic, physical, chemical or other type of environmental perturbations? Or formally, given a specific dynamical mapping **x**(*t* + δ*t*) = **F**(**x**(*t*), **u**, δ*t*), and its associated state space, how can we study the conditions under which a transition event occurs among the attractor states x^*^? Is there a reproducible pattern of transitions? Can we alter the expected pattern through specific external control perturbations **u**? To what extent are the observed robust and altered temporal or spatial morphogenetic patterns emergent consequences of the GRNs? The extension of GRN dynamical models and their analysis in order to address these and similar questions has shown to be a fruitful area of research in recent years (Han and Wang, 2007; Alvarez-Buylla et al., 2008; Wang et al., 2010b, 2014; Choi et al., 2012; Qiu et al., 2012; Villarreal et al., 2012; Zhou et al., 2012; Li and Wang, 2013; Zhu et al., 2015). The conceptual basis for most of these efforts is the EAL.

### 1.3. Deterministic EAL models from genetic circuits

#### 1.3.1. An introductory toy model

A quite simple auto-activating single-gene circuit, a basic model of cell differentiation induction, is exposed in Ferrell (2012) as a conceptual tool to discuss some difficulties regarding Waddington's EL. In this work an EAL is mathematically described by a potential function. In dynamical systems theory, besides the state space approach explained briefly above, there is another way to visualize the dynamics of a system, but applicable only if the system is simple enough: the potential function (Strogatz, 2001; Fuchs, 2013a). The potential is a function *V*(*x*) which (in one-dimensional systems) fulfills the relation given by:

(2) $$\frac{dx}{dt}=f(x)=-\frac{dV(x)}{dx},$$

i.e., *f*(*x*) is the negative derivative of the potential, which can be found by direct integration:

(3) V(x)=−∫f(x)dx.

**Figure 2:**
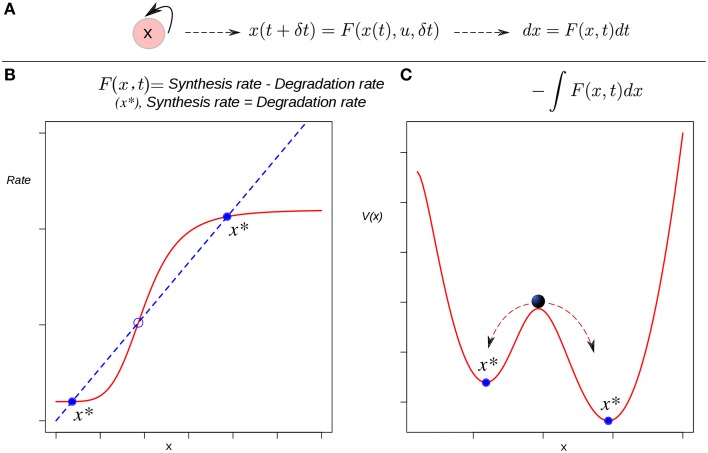
**The derivation of a potential function to visualize the epigenetic landscape and the dynamics of a system. (A)** The causal connection between the state of the system (an auto-activating gene circuit) at a certain time and its state at a later time is modeled by a differential equation. **(B)** Attractors in rate-balance analysis. The blue dotted line and the red curve represent respectively a linear degradation rate, and a non-linear synthesis rate for the circuit's gene; the restrictions imposed by the circuit to the systems dynamics are met when both rates are balanced. The states that meet this balanced condition are stationary, and if stable (filled circles), are denoted as attractor states x^*^. Circles represent stationary states. **(C)** The potential function. The attractor states x^*^ lie at the bottoms of valleys (minima). The trajectories starting from unstable, transitory states are attracted by the minima of the potential. The relative stability of the left (right) attractor with respect to the other is lower (higher) as quantified by the lower (higher) barrier height between them.

Such a function defines an attractor landscape for the given dynamical system, and its plot graphically represents the dynamics of the system (Figure 2). Specifically, *minima* of the potential correspond to fixed-point attractors (e.g., cell types), and *maxima* correspond to unstable fixed-points. The motion, i.e., the state trajectories are given by the gradient lines (the lines of steepest descent of the potential). The trajectories are attracted by the minima of the potential. This corresponds to an intuitive, direct derivation of the EAL: a “height” value is associated to each of the points in the state-space in a way that those regions corresponding to attractors will have a lower value than that of the other transitory states (Figure 2C). Conceptually, the rolling ball of Waddington's EL will represent the state of a differentiating cell moving from higher to lower regions in state space. Thus, the calculated heights of the different attractors are expected to reflect their developmental potential in a hierarchical way: the lower height the lower potential for differentiation.

All one-dimensional systems have a potential function, but most two- or higher-dimensional systems do not (Fuchs, 2013a). This means that one could only apply this method if the cell is represented by a single-gene (single variable) circuit. Furthermore, note that here the EAL plays the role of a “toy” model useful in conceptual discussions, a role quite relevant (see Ferrell, 2012) but similar to that of the original metaphorical proposal of Waddington. In this review we devote more attention to the application of EAL models to real specific developmental processes with explanatory and predictive purposes that generally involve n-dimensional GRN. Thus, a more “realistic” sub-network model incorporating several transcription factors in a modular structure is necessary in such cases. The application of the integration-based potential function approach, however, cannot be applied to cases with a higher number of genes. Also, one should be cautious when postulating the existence of a potential for living systems in strict sense: a cell is an open non-equilibrium thermodynamical system, and its dynamics in general does not follow a gradient (since the transition rate between two given attractor states is not path-independent). For details, see (Zhou et al., 2012; Huang, 2013). For this reason authors use the term “quasi-potential” when speaking about cellular dynamics from a system-dynamics point of view (see below).

In the general case, the dynamics of continuous-time models of GRNs is given by more general types of autonomous differential equations (DEs). The time evolution of the cell state **x**(*t*) is commonly modeled by the system of DEs:

(4) $$\frac{dx_{i}(t)}{dt}=F_{i}(x_{1},x_{2},\ldots ,x_{f},u),$$

where *i* = 1, 2, …, *n* for a GRN of *n* genes. A dynamics defined by such a general DE is a special form of the map in Equation (1). In general, the functions **F** in the continuous-time model for cellular dynamics (Equation 4) are non-linear, and cannot be analytically integrated and derived from a gradient. Numerical approaches have been proposed to draw a deterministic “quasi-potential” for two-gene circuits (see, for example Bhattacharya et al., 2011). In what follows we focus on medium size GRN modules, where neither the direct integration nor the numerical deterministic approach are applicable. We start with the simplest models of GRN dynamics.

### 1.4. Stochastic EAL models from boolean GRNs

The first computational model envisioned for the simulation and analysis of the dynamic behavior of GRNs was the Boolean Network (BN) model (Kauffman, 1969, 1993). This model has been extended to model various developmental processes in the context of the EAL (Han and Wang, 2007; Alvarez-Buylla et al., 2008; Ding and Wang, 2011; Choi et al., 2012; Flöttmann et al., 2012). A BN models a dynamical system assuming both discrete time and discrete state. This is expressed formally with the mapping:

(5) $$x_{i}(t+1)=F_{i}(x_{1}(t),x_{2}(t),\ldots ,x_{f}(t)),$$

where the set of functions *F_i_* are logical propositions expressing the relationship between the genes that share regulatory interactions with the gene *i*, and where the state variables *x_i_*(*t*) can take the discrete values 1 or 0 indicating whether the gene *i* is active or not at a certain time *t*, respectively. An experimentally grounded Boolean GRN model is then completely specified by the set of genes proposed to be involved in the process of interest and the associated set of logical functions derived from experimental data (Azpeitia et al., 2014). A dynamics defined by such a mapping is a special form of the map in Equation (1).

#### 1.4.1. Attractor transition probability approach to explore the EAL

As stated above, in a deterministic framework, once a cell state corresponds to an attractor, it will remain there indefinitely. The set of conditions that lead to each attractor comprise the attracting *basin*. Under stochastic fluctuations, the borders of attractor regions in state space may be reached and may be crossed, leading to transitions from one attractor to another one (Ebeling and Feistel, 2011). Thus, the implementation of an stochastic dynamical model opens the opportunity to study signal-independent transitions among attractors. There are several approaches to include stochasticity in dynamical models. One approach is based on the idea of introducing transition probabilities. As discussed above, when studying cellular developmental dynamics, the transitions of interest are those among attractor states. Can these transitions be studied in terms of probabilities? Indeed, since Boolean GRN can be extended to include stochasticity and transition probabilities among attractors can then be estimated. Several ways to include stochasticity in a Boolean GRN model have been proposed (Garg et al., 2012). One way is the so-called stochasticity in nodes (SIN) model. Here, a constant probability of error ξ is introduced for the deterministic Boolean functions. In other words, at each time step, each gene “disobeys” its Boolean function with probability ξ. Formally:

(6) $$\begin{array}{l} P_{x_{i}(t+1)}[F_{i}(x_{reg_{i}}(t))]=1-\xi ,\\ P_{x_{i}(t+1)}[1-F_{i}(x_{reg_{i}}(t))]=\xi . \end{array}$$

The probability that the value of the now random variable *x_i_*(*t* + 1) is determined or not by its associated logical function *F_i_*(**x***_reg_i__*(*t*)) is 1 − ξ or ξ, respectively.

**Figure 3:**
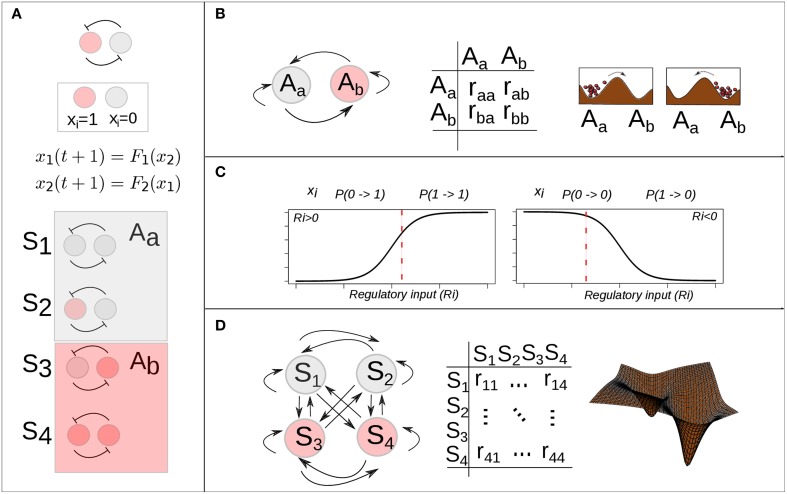
**Stochastic epigenetic landscape models from Boolean dynamics. (A)** A simple mutual-inhibition circuit is modeled as a Boolean network: discrete temporal evolution and binary (0, 1) state variable. The discrete state space corresponds to the set of binary vectors (here 4 possible states) and is partitioned by two basins of attraction. **(B)** There are 4 (2^2^) possible transitions among the two attractors. A 2 × 2 transition probability matrix specifies the probability of each possible transition. **(C)** Han and Wang proposed the use of a sigmoidal function of the total regulatory input (Ri) to calculate the probability of a one-step state transition of one gene *i* (Han and Wang, 2007). A specific value of the function (vertical red dotted line) gives the probability of the gene *i* becoming active (left) or inactive (right), given its regulatory input (Ri) in the current time. **(D)** There are 16 (2^2^ × 2^2^) possible transitions among the 4 (2^2^) possible states. A 2^2^ × 2^2^ transition probability matrix specifies the probability of each possible transition.

Alvarez-Buylla and collaborators used this extended BN model to explore the EAL associated with an experimentally grounded GRN (Alvarez-Buylla et al., 2008) (see below). In a BN model the set of possible states is finite. Specifically, due to its binary state character the state space of a Boolean GRN with *n* genes has a size of 2^*n*^ and is composed by the set of all possible binary vectors of length *n* (see Figure 3A). By simulating a stochastic one-step transition, according to the model in Equation (6) and the mapping in Equation (5), and starting from each of all the possible states in the system for a large number of times, it is possible to estimate the probability of transition from an attractor *i* to an attractor *j* as the frequency of times the states belonging to the basin of the attractor *i* are mapped into a state within the basin of the attractor *j*. For detail see (Azpeitia et al., 2014). In Alvarez-Buylla et al. (2008), the authors followed this simulation approach to estimate a transition probability matrix Π with components:

(7) $$\pi_{ij}=P(A_{t+1}=j|A_{t}=i),$$

representing the probability that an attractor *j* is reached from an attractor *i* (Figure 3B). Once the set of attractors is known and the transition probability matrix is estimated, it is straightforward to implement a discrete time Markov chain model (DTMC) and obtain a dynamic equation for the probability distribution (for details, see Allen, 2010):

(8) $$P_{A}(t+1)=\Pi P_{A}(t),$$

where *P_A_*(*t*) is the probability distribution over the attractors at time *t*, and Π is the transition probability matrix previously estimated. This equation can be iterated to simulate the temporal evolution of the probability distribution over the attractors starting from a biologically meaningful initial distribution. The extension of a Boolean GRN in order to apply this approach is quite simple and intuitive; however, there is a limitation that impedes its general applicability: as the size of the GRN grows, it becomes difficult to exhaustively characterize the attractor's landscape associated with the GRN in terms of the emergent attractors and its corresponding basins of attraction. If the dynamics of the Boolean GRN is not exhaustively characterized, the corresponding transition probabilities among attractors cannot be estimated using the proposed approach. Additionally, other implementations of stochasticity within BN models have been discussed (Garg et al., 2012). Additional examples should be worked out with such various approaches to test which is more practical and if all yield equivalent results.

#### 1.4.2. Probabilistic landscape (quasi-potential) approach

Han and Wang proposed a different approach in order to extend a BN model. Their goal was to first estimate the one-step transition probabilities among all the possible states in the state space and not just among given attractors (Han and Wang, 2007). For this, they implemented a variation of the BN that was previously proposed by Li and collaborators (Li et al., 2004) and which has been called the threshold network formalism (Thompson and Galitski, 2012). In this model, the structure of the network is formally represented with an adjacency matrix **C**, whose components *c_ij_* indicating the nature and strength of the interaction from the gene *j* to gene *i*. The dynamic mapping for this BN model takes the form:

(9) $$x_{i}(t+1)=\left\{ \begin{array}{l} 1,\;\sum_{j}c_{ij}x_{j}(t)+b_{i}>0,\\ 0,\;\sum_{j}c_{ij}x_{j}(t)+b_{i}<0,\\ x_{i}(t),\;\sum_{j}c_{ij}x_{j}(t)+b_{i}=0, \end{array}\right.$$

where *b_i_* is a parameter representing the ground state of the gene *i*: its state in the absence of regulation. The set of parameters (i.e., *b_i_* and *c_ij_*) can be chosen to force the dynamics of the BN to be consistent with those of a BN with a specific set of logical propositions (for details, see Supplementary Material in Choi et al., 2012). The mapping in Equation (9) can be conceptualized as follows: if the total input of a gene in the network is positive (activation), negative (repression) or zero; the future state of the gene will be active, inactive or unchanged from its previous state, respectively. Here, the total input of a gene is the sum of the previous states of the genes regulating it. The characterization of the entire attractor's landscape can then be done through numerical iterations of this dynamical map as long as the network has a moderate size.

Han and Wang extended the deterministic BN model into a probabilistic framework by introducing a transition probability matrix. However, if the interest is focused on the computation of the probability of transition from one state to another state for each of the 2^*n*^ possible phenotypes in state space, then it is necessary to introduce a transition probability matrix with the probability of all possible transitions and not just among attractors. In order to make such computation feasible, Han and Wang introduced a simplification: they assumed that the one-step transition probability of one state to another can be expressed as the product of the probability of each gene in the network being activated or not, given the state of the network in the previous time (for details, see Han and Wang, 2007, and Supplementary Material in Choi et al., 2012). Formally:

(10) $$\pi_{kj}=P(x(t+1)=k|x(t)=j)=\prod_{i=1}^{n}P(x_{i}(t+1)|x(t)=j),$$

where *j* and *k* represent two different cell states and can take values from [1, …, 2^*n*^]; *n* is the number of genes in the network. The factorized transition probabilities are calculated by inserting a non-zero regulatory input ($\sum_{j=1}^{n}c_{ij}x_{j}(t)+b_{i}(t)\neq 0$) as the argument of a sigmoidal function whose range spans from 0 to 1, which is to say:

(11) $$P(x_{i}(t+1)=1|x(t)=j)=\frac{1}{2}\pm \frac{1}{2}\tanh\left[\mu \sum_{j=1}^{n}c_{ij}x_{j}+b_{i}\right].$$

In the case of no input (i.e., $\sum_{j=1}^{n}c_{ij}x_{j}(t)+b_{i}=0$) a small-valued parameter *d* is introduced:

$$P(x_{i}(t+1)=x_{i}(t)|x(t)=j)=1-d.$$

Hence, in this approach, the probability that a gene *i* will be active (1) at a future time *t* + 1 will be closer to one as long as its total input at the previous time *t* is high. Similarly, the probability of being inactive (0) at the future time will be closer to 1 as long as the regulatory input is low (see Figure 3C). On the other hand, if there is no input to the gene, the probability of no change from its previous state is close to 1, and the closeness depends on the parameter *d*, a small number representing self-degradation. Intuitively, these rules ensure that the state of a gene will flip only if its total input is large enough.

After having calculated these probabilities, the general idea is then to use this information to obtain an appropriate “height” measure for each of the 2^*n*^ states. With this in mind, the interest is first in calculating a steady-state probability distribution *P_SS_*(**x**). This stationary probability distribution is analogous to stationary configurations in the deterministic case; however, in the stochastic framework, the probability of being in any particular state, rather than the state of the system, is what is kept invariant along time. In other words, when this stationary distribution is reached, the probability of observing a cell in a particular state does not change. Intuitively, one would expect that attractors would have a higher probability of being reached than transitory states. Thus, from a landscape perspective, the potency of differentiation and height should be inversely related with the probability. The approach that has been followed is to associate this *P_SS_*(**x**) with a height value. Wang has proposed that the probability distribution for a particular state *P*(**x**_*i*_) = exp[*U*(**x**_*i*_)], and from this expression then *U*(**x**_*i*_) = − ln *P*(**x**_*i*_), where *i* = 1, …, 2^*n*^. This function *U* has been termed the (probabilistic) quasi-potential (Huang, 2009, 2012; Wang et al., 2010b)]. How are the “quasi-potential” and the steady-state probability formally related to each other is still an open research area (Zhou et al., 2012) (see below).

The key point which has been emphasized by Wang and coworkers is that, although there is (in general) no potential function directly obtainable from the deterministic equations for a given network, a generalized potential (or “quasi-potential”) function can be constructed from its probabilistic description. This generalized potential function is inversely related to the steady-state probability (Wang et al., 2006; Han and Wang, 2007; Lapidus et al., 2008). For the case of the extended BN model, once the transition matrix is calculated, the information of the steady-state probabilities can be obtained by solving a discrete set of master equations (ME) for the network (Han and Wang, 2007). The so-called ME is a dynamical equation for the temporal evolution of a probability distribution (for details, see Haken, 1977; Gardiner, 2009). In discrete form it is written as:

(12) $$\frac{\partial }{\partial t}P(x_{i})=\sum_{j}W_{ji}P(x_{j})-\sum_{j}W_{ij}P(x_{i}),$$

where we used *W_ij_* to denote the transition probabilities resulting from Equation (11). The difference between this dynamical equation and the one discussed in the previous section is that here the time variable is treated as a continuous one. In general, it is quite complicated to analyze MEs. In the case of this model, one ME is obtained for each of the 2^*n*^ states. Han and Wang propose to analyzed the whole set of equations following a numerical (iterative) method starting from uniform initial conditions *P*_**x**_*i*__(*t*_0_) = 1/2^*n*^ and iterating the system until a stationary distribution is reached (Han and Wang, 2007).

### 1.5. Stochastic EAL models from continuous GRNs

As in the case of the deterministic BN model revised above, a general deterministic system of DEs used to describe a GRN can be extended in order to include stochasticity. Such continuous models may be more appropriate to approach certain biological processes. The most intuitive extension considers the introduction of driving stochastic forces. In this approach, Equation (4) is extended to:

(13) $$\frac{dx_{i}(t)}{dt}=F_{i}(x,u)+\xi_{i}(t),$$

where ξ_*i*_(*t*) is the *i*th component of a driving stochastic force with zero mean value (i.e., < ξ_*i*_(*t*) >= 0). This description, the so-called Langevin equation, is frequently used to model cellular dynamics under stochastic fluctuations (Hoffmann et al., 2008; Wang et al., 2010b; Villarreal et al., 2012; Li and Wang, 2013).

**Figure 4:**
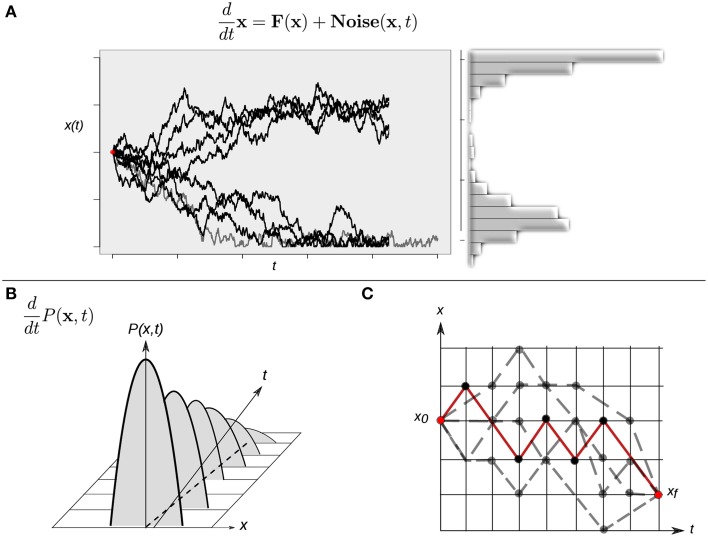
**Different approaches to study continuous-time stochastic models of the epigenetic landscape and developmental dynamics. (A)** A continuous-time stochastic (diffusion) model is driven by a drift (deterministic) component **F** and an stochastic (**Noise**) force. The graph shows 10 different realizations of the stochastic dynamics of the same, single cell starting form exactly the same initial condition (red dot). This realizations perspective corresponds to the Langevin equation description. The right histogram represents an approximation of the corresponding distribution over the realizations. **(B)** The picture represents the time evolution of a hypothetical probability distribution. A population of cells initially presents a narrow distribution centered at an intermediate state value: most cells have an intermediate state and no individuals show an extreme (low or high) value. As time evolves the shape of the distribution changes—gets wider—, and the population reaches lower and higher values. This perspective corresponds to the Fokker-Planck equation description. **(C)** A cell can follow different paths (gray dotted lines) to reach a final state *x_f_* starting from an initial state *x*_0_. A finer quantitative characterization of the specific transition from state *x*_0_ to state *x_f_* in terms of highly probable paths and difficulty of differentiation processes can be gained by means of calculating a dominant path (red line) for the transition using a path-integral formalism. For simplicity, the cell state is represented by one variable *x* in all three cases.

Although intuitively simple at first sight, the consideration of a randomly varying quantity affecting the dynamics of the system implies several conceptual issues that should be considered in some detail. Any single cell will follow an erratic trajectory in state space, and its developmental dynamics will make each realization different *even if it starts from exactly the same initial condition*. Under this stochastic scenario, two equivalent perspectives to study the stochastic dynamics can be considered. On the one hand, the analysis could be focused on trajectories described by Langevin-type equations, which describe the developmental dynamics of a single cell (Figure 4A). On the other hand, as the stochastic forces ξ_*i*_(*t*) vary from cell to cell in an *ensemble* (population) of cells, the state **x**(*t*) will also vary from cell to cell at any given time. One therefore may ask for the probability *P*(**x**, *t*) to find the state of a cell in a given state interval of the state space or, equivalently, for the frequency of cells in the ensemble whose states are in that state interval. In the latter situation, the focus shifts from the dynamics of the state of one cell to the dynamics of the distribution over the states in a given ensemble of cells. Indeed, an equation for the temporal evolution of this distribution *P*(**x**, *t*) can be constructed, and this corresponds to the so-called *Fokker-Plank equation (FPE)*:

(14) $$\frac{\partial P}{\partial t}=-\sum_{i}\frac{\partial }{\partial x_{i}}[A_{i}(x)P]+\frac{1}{2}\sum_{i,j}Q_{i,j}(x)\frac{\partial^{2}}{\partial x_{i}\partial x_{j}}P.$$

In mathematical terms, the corresponding process is known as a *diffusion process*, a mathematical model for stochastic phenomena evolving in continuous time; the vector **A**(**x**) is known as the drift vector and the matrix **Q**(**x**) as the diffusion matrix (for details, see Risken, 1984; Gardiner, 2009; Fuchs, 2013b). The FPE describes the change of the probability distribution of a cell state during the course of time (Figure 4B). Conceptually, the latter modeling perspective can be interpreted as the temporal evolution of a cloud (ensemble) of cells diffusing across the state space following both attracting and stochastic forces (see Huang, 2010 for a conceptual perspective).

The stochastic nature of the trajectories also produce qualitatively richer dynamics in state space. For example, if one is interested in the developmental connection between one specific initial cell state and one specific final cell state—for example, two different given attractors—there is no longer a single possible path connecting them. Instead, the same final cellular phenotype can be reached following different paths in state space (Figure 4C). This situation raises yet additional interesting issues: are all the paths equally probable? Is there a dominant path for such a transition from one attractor to another one? Physicists have proposed the so-called path-integral formalism in order to tackle these and similar questions (Wio, 1999). Specifically, one may want to answer what is the probability of starting from an initial cellular phenotype at a certain time and ending in another cellular phenotype at a future time. The conceptual basis of this strategy is based on the idea of calculating an average trajectory (e.g., integrating over the possible paths). The calculated averaged path corresponds to the dominant path that the underlying process is expected to preferentially follow (Figure 4C).

Given the intuitive appeal of a landscape perspective to general dynamics, the existence of a potential or “potential-like” function associated with diffusive systems has been an intensive focus of study in theoretical physics and applied mathematics. Ao and co-workers have proposed a transformation that allows the definition of a function *U*(**x**) which successfully acquires the dynamical meaning of a potential function. The corresponding approach has been applied successfully to study several biological systems such as the *phage lambda life cycle* (Zhu et al., 2004), and the carcinogenesis processes, Ao et al. (2008), Wang et al. (2013, 2014), and Zhu et al. (2015) from a landscape perspective. This transformation has also been discussed recently in the context of general methods for the decomposition of multivariate continuous mappings *F*(**x**) and their associated quasi-potentials (Zhou et al., 2012). From the available decomposition methods, the one that has been applied the most to specific developmental processes is the potential landscape and flux framework proposed by Wang et al. (2008). In this framework, the continuous dynamical mapping *F*(**x**) is decomposed into a gradient part and a flux, curl part (for details, see Wang, 2011). This approach has been applied, for example, to the study of the yeast cell cycle [Wang et al. (2006, 2010a)]; a circadian oscillator (Wang et al., 2009); the generic processes of stem cell differentiation and reprogramming (Wang et al., 2010b; Xu et al., 2014); and neural differentiation (Qiu et al., 2012). Recently, this method has been applied in the context of the differentiation and reprogramming of a human stem cell network (Li and Wang, 2013). Here we further discuss the latter as a diffusion landscape approach to study stem cell differentiation. Although the technical details of decomposition methods for diffusive systems from a landscape perspective are out of the scope of the present review, we point the reader to Ao (2004), Kwon et al. (2005), Yin and Ao (2006), Ao et al. (2007), Ge and Qian (2012), Zhou et al. (2012), and Lv et al. (2014) for further details.

To summarize this section: when a stochastic component with specific properties is introduced in a continuous-time dynamical model of developmental dynamics, the behavior of the system can be studied from different, mathematically equivalent perspectives. One of the perspectives could be more appropriate than the others, given the biological question of interest; the different perspectives complement each other, nonetheless. It is important to note that the three approaches mentioned above (e.g., Langevin, FPE, and path-integral) although just recently introduced in systems biology (Wang et al., 2010b, 2011; Villarreal et al., 2012; Zhang and Wolynes, 2014; Wang et al., 2014); are actually well-established tools in non-equilibrium statistical mechanics and the stochastic approach to complex systems (Haken, 1977; Lindenberg and West, 1990; Gardiner, 2009).

### 1.6. From EAL models to biological insights

#### 1.6.1. EL exploration in flower morphogenesis

Alvarez-Buylla and collaborators applied the attractor transition probability approach (Equations 5–8 and Figure 3B) to explore the EAL explained above in order to study flower patterning shared by most angiosperms or flowering species (Alvarez-Buylla et al., 2008). In flowering plants, a floral meristem is sequentially partitioned into four regions from which the floral organ primordia are formed and eventually give rise to sepals in the outermost whorl, then to petals in the second whorl, stamens in the third, and carpels in the fourth whorl in the central part of the flower. This spatiotemporal pattern is widely conserved among angiosperms. Can the temporal pattern of cell-fate attainment be explained by the interplay of stochastic perturbations and the constraints imposed by a non-linear GRN? Starting from the previously characterized Boolean GRN of organ identity genes in the *A. thaliana* flower (Espinosa-Soto et al., 2004), and applying the stochastic approach described in Equations (5–8), the authors showed that the most probable order in which the attractors are attained is, in fact, consistent with the temporal sequence in which the specification of corresponding cellular phenotypes are observed *in vivo*. The model provided, then, a novel explanation for the emergence and robustness of the ubiquitous temporal pattern of floral organ specification, and also allowed predictions on the population dynamics of cells with different genetic configurations during development (Alvarez-Buylla et al., 2008). Note that in this approach, through the calculation of transition probabilities among attractors, it is possible to explore the EAL associated with a GRN. It also constitutes a new approach to understanding a morphogenic process and also implies that GRN topologies could have, in part, evolved in response to noisy environments. In the same contribution, the authors also showed that a stochastic continuous approximation of the GRN under analysis yielded consistent results. Importantly, in this study it was argued that the fact that observed patterns of cell-fate transitions could be significantly constrained by GRN in the context of noisy perturbations does not excludes the relevance of deterministic signals.

#### 1.6.2. From probabilistic landscapes to putative cancer therapies

The probabilistic landscape (quasi-potential) approach has been applied to two specific processes: cell cycle regulation (Han and Wang, 2007), and DNA damage response (Choi et al., 2012). In the former case, the focus was on the global robustness properties of the network. Here we discuss the biological implications derived from the latter case. Choi and collaborators applied this BN probabilistic landscape approach (Equations 9–12 and Figures 3C,D) to study state transition in a simplified network of the p53 tumor suppressor protein. The analysis of this network from an EAL perspective allowed the systematic search for combinatorial therapeutic treatments in cancer (Wang, 2013). Given the network, key nodes and interactions that control p53 dynamics and the cellular response to DNA damage were identified by conducting single node and link mutation simulations; as a result, one network component, the molecule Wip1, was identified as one of the critical nodes. The flexibility of the BN model also enabled the specification of a MCF7 cancer cell by fixing the state of three nodes of the “normal” network in the course of simulations (for details, see Choi et al., 2012; Wang, 2013). Having specified two different network models, it was possible to compare the dynamics and associated quasi-potential of both normal and cancer cells in the absence and presence of DNA damage. Previous experimental observations indicated that prolonged p53 activity induces senescence or cell death; this behavior was shown to result from the inhibition of the interaction between the molecules Mdm2 and p53 caused by the action of the small molecule Nutlin-3 (Purvis et al., 2012). Using the model, Choi and collaborators predicted that neither Wip1 nor Mdm2-p53 interaction mutation alone were sufficient to induce cell death for MCF7 cancer cells in the presence of DNA damage; furthermore, the model provided a mechanistic explanation for this behavior: the effect of each of this perturbations alone is not enough to move the system out of an specific attractor's basin. But the simultaneous application of the two perturbations may drive cancer cells to cell death or cell senescence attractors. These theoretical predictions were then validated using single-cell imaging experiments (Choi et al., 2012; Wang, 2013).

This study illustrated in an elegant way how cancer therapeutic strategies can be studied in mechanistic terms using a computational EAL model. It must be pointed out that this result opened the door to the rational design of system dynamics cancer therapeutical techniques, in contrast to trial and error and reductionist approaches that have dominated the biomedical field up to now (Huang and Kauffman, 2013).

#### 1.6.3. A diffusion approach to study the EAL

The three perspectives to study continuous-time stochastic models of developmental dynamics briefly described above and represented in Figure 4 have been applied to understanding actual developmental cases from an EAL point of view. For example, Villarreal and collaborators recently proposed a procedure to construct a probabilistic EAL by calculating the probability distribution of stable gene expression configurations arising from the topology of a general N-node GRN (Villarreal et al., 2012). In this approach, the focus of study is the temporal evolution of the distribution over state space (Equation 14 and Figure 4B) starting from a position centered on a specific attractor configuration. Intuitively, the proposed framework predicts how a cloud of cells distributed over a particular attractor will diffuse in time to the neighboring regions (attractors) in state space, given a specific GRN (which constraints the state trajectories). The method has been applied to the case of early flower morphogenesis (see subsection above); and its behavior, in both wild type and mutant conditions. The authors recovered patterns that are in agreement with the temporal developmental pattern of floral organs attainment in *A. thaliana* and most flowering species (Alvarez-Buylla et al., 2008; Villarreal et al., 2012). The AEL perspective has recently also given important insights into the problem of carcinogenesis trough the quantitative implementation of the *molecular–cellular network hypothesis* by Ao and co-workers (for details, see Wang et al., 2014; Zhu et al., 2015).

#### 1.6.4. Cell fate decisions in the human stem cell landscape

Recently, Li and Wang adopted the diffusion approach to study a previously published human stem cell developmental network (see Chang et al., 2011) composed of 52 genes (Li and Wang, 2013). In this study they showed how the three perspectives represented in Figure 4 can complement each other in the study of cellular differentiation: (1) through the numerical analysis of the Langevin-like equations for the complete network they acquired a landscape directly from the statistics of the trajectories of the system (Equation 13 and Figure 4A); (2) by means of approximations they studied the evolution of the probabilistic distribution and obtained an steady-state distribution (Equation 14 and Figure 4B); and (3) using the path-integral formalism (Figure 4C) they calculated the dominant paths (Wang et al., 2011). The obtained paths were interpreted as the biological paths for differentiation and reprogramming (Li and Wang, 2013). As Li and Wang showed, from the results of the three perspectives it is possible to quantitatively describe the underlying EAL. One then may be interested in how the EAL changes in response to specific perturbations.

A general question in stem cell research concerns the underlying mechanisms that explain the known reprogramming strategies, which commonly consist on combining perturbations to specific transcription factors. Li and Wang systematically tested which genes and regulatory interactions imply the greatest alterations to the quantitative properties of the EAL (e.g., height values and transition rates) when perturbed. Interestingly, several biological observations associated with the manipulation of the so-called Yamanaka factors (Oct3/4, Sox2, Klf4, c-Myc)—the transcription factors considered the core regulators in the induction of pluripotency—were consistent with the observed modeling results. For example, simulated knockdown perturbations to these factors consistently increased (lowered) the probability (height) of the differentiation state. On the other hand, the path-integral formalism allowed them to show how specific perturbations to these factors cause the differentiation process to be easier or harder in terms of the time spent during transitions and the characteristics of the differentiation paths. Overall, this study presented an important contribution toward the mechanistic, dynamical explanation of the characterized reprogramming strategies in terms of the properties of the underlying EAL.

### 1.7. Concluding remarks

An overall strategy for the practical implementation of what we call EAL models comprises four steps: (1) establishment of an experimentally grounded GRN; (2) characterization of the attractor (and quasi-potential) landscape through dynamical modeling; (3) computational prediction of cell state responses to specific perturbations; and (4) analysis of the prevailing paths of cell fate change. The first step (1) is already a well-established research problem that includes expert curation of experimental data and/or statistical inference. In this review we focused on the second step and presented examples of how steps (3) and (4) can be achieved once a EAL model is effectively constructed. As shown here, there are several ways to implement an EAL model starting from a GRN. The specific choice should be made considering the properties of the network and the associated questions of interest.

The methodologies reviewed here are mostly well-suited to approach the problem of differentiation and temporal cell-fate attainment in a mechanistic setting. The observed behavior results from constraints given by the joint effect of non-linear regulatory interactions and the inherent stochasticity prevalent in GRN. The actual physical implementation of these generic mechanisms in a multicellular system would necessarily imply additional sources of constraint and spatially explicit, multi-level modeling platforms. Tissue-level patterning mechanisms such as cell-cell interactions; chemical signaling; cellular growth, proliferation, and senescence; in addition to mechanic and elastic forces at play in cells, tissues and organs, inevitably impose physical limitations which in turn affect cellular behavior. This would thus imply non-homogenous GRNs with contrasting additional chemical and physical constraints, that in a cooperative manner underlie the emergence of positional information and morphogenetic patterns. Given this fact, the next logical step to extend EAL and associated dynamical models would be to account for these physical processes in an attempt to understand how cellular decisions occur during tissue patterning and not just in cell cultures. Although some progress has been presented in this direction (see, for example Barrio et al., 2010, 2013), the problem remains largely open, specially in terms of explicitly considering the constrains imposed by the underlying GRN and EAL.

From a theoretical perspective, a further challenge would be to carefully evaluate the assumptions implicit in the EAL models. For example, the adoption of the diffusive perspective briefly explained above—which is often taken as a standard in stem cell systems biology—implicitly assumes certain properties about the forces driving the temporal evolution of the system (Lindenberg and West, 1990). Are these conditions universally met by developmental systems? Recent interesting work is starting to suggest the biological relevance of additional constraints such as state-dependent fluctuations (Pujadas and Feinberg, 2012; Weber and Buceta, 2013), as well as time-dependent dynamical behavior (Mitra et al., 2014; Verd et al., 2014). In both cases, a dynamically changing EAL is proposed as a potentially more accurate description of developmental processes than its static counterpart.

Overall, the application of the methodologies discussed in this review to specific developmental processes has shown the practical relevance of dynamical models consistent with the conceptual basis of the classical EL and the fundamental role of the constraints imposed by the GRN interactions. The different EAL modeling approaches are useful to answer specific questions and can complement each other. So far, EAL models have shown to be an adequate framework for understanding stem cell differentiation and reprogramming events in mechanistic terms; and are also starting to show promise as the basis for rational cancer therapeutic strategies, as well as other interesting issues in developmental biology and evolution.

### Conflict of interest statement

The authors declare that the research was conducted in the absence of any commercial or financial relationships that could be construed as a potential conflict of interest.
